# Senescence and associated blood–brain barrier alterations in vitro

**DOI:** 10.1007/s00418-021-01992-z

**Published:** 2021-05-27

**Authors:** Ellaine Salvador, Malgorzata Burek, Mario Löhr, Michiaki Nagai, Carsten Hagemann, Carola Y. Förster

**Affiliations:** 1 Department of Anaesthesia and Critical Care, University Hospital Würzburg, Würzburg, Germany; 2 Department of Neurosurgery, Tumorbiology Laboratory, University Hospital Würzburg, Würzburg, Germany; 3 Department of Cardiology, Hiroshima City Asa Hospital, Aaskita-ku, Hiroshima, Japan

**Keywords:** Senescence, Blood–brain barrier, In vitro model, Aging, CNS diseases

## Abstract

Progressive deterioration of the central nervous system (CNS) is commonly associated with aging. An important component of the neurovasculature is the blood–brain barrier (BBB), majorly made up of endothelial cells joined together by intercellular junctions. The relationship between senescence and changes in the BBB has not yet been thoroughly explored. Moreover, the lack of in vitro models for the study of the mechanisms involved in those changes impede further and more in-depth investigations in the field. For this reason, we herein present an in vitro model of the senescent BBB and an initial attempt to identify senescence-associated alterations within.

## Introduction

Majorly composed of endothelial cells, the blood–brain barrier (BBB) plays a pivotal role in maintaining central nervous system homeostasis. Breakdown of the BBB is a key feature of neuroinflammatory conditions and associated with the influx of inflammatory cells, fluids and proteins, including complement and cytokines (Rubin and Staddon 1999; D’Atri and Citi 2002; Förster 2008). When the BBB is compromised, homeostatic breakdown may occur, leading to degenerative effects on BBB function. In a disrupted BBB, molecules which do not usually permeate the barrier, leak through it. This is majorly attributed to the loss of BBB integrity usually imparted by tight junctional sealing (Lockhead et al. 2020). Among the various regulators of barrier integrity is claudin-5, a major tight junction protein expressed in brain microvascular endothelial cells (Jia et al. 2014; Luissint et al. 2012; Burek et al. 2010). In addition, adaptor proteins acting as cytoskeletal linkers such as zonula occludens-1 (ZO-1) are important components of the brain endothelial barrier and function as regulators of tight junction assembly (Zihni et al. 2016; Fanning and Anderson 2009). Prior to formation of TJs, cell-to-cell contacts need to be initiated and this is carried out by adherens junctions (AJs). AJs play an important role in the control of vascular permeability (Giannotta et al. 2013). VE-Cadherin, the main integral membrane protein of endothelial AJs, links AJs to actin cytoskeleton as well as controls endothelial cell survival and stabilization of blood vessel assembly (Crosby et al. 2005; Carmeliet et al. 1999). Overall, it has been demonstrated both in the lab and by computational simulations that mutational changes of BBB tight junction proteins and transporters bring about detrimental effects to the brain (Shityakov and Förster 2018).

Considering that the mammalian brain requires a constant supply of glucose as its main source of energy for the regulation of brain physiology, one major role of endothelial cells is the transport of glucose (Mergenthaler et al. 2013). The glucose transporter GLUT-1 is the main glucose transporter in brain endothelial cells and is essential for maintaining normal neurological functions (Patching 2017). Lower GLUT-1 levels have been associated with the impairment of microvasculature as well as with BBB dysfunction in patients with Alzheimer’s disease (AD), which is closely associated with senescence (Winkler et al. 2015). In fact, GLUT-1 expression was significantly decreased in patients with AD compared to healthy controls (Vogelsang et al. 2018). Moreover, GLUT-1 immunoreactive structures are significantly decreased with age in gerbils and mice brain (Lee et al. 2018).

Resulting from a variety of stresses and irreversible growth arrest is cellular senescence, a stress response linking the degenerative and hyperplastic pathologies of aging. Although cellular senescence is primarily involved in normal development, cell plasticity, and tissue repair, when the cellular program regulating these processes is disturbed, disease, and aging ensue (Rhinn et al. 2019). Senescence results in DNA damage, telomere shortening, telomere dysfunction, and oncogenic stress, which give rise to dysfunctional, transformed, or aged cells (Mohamad Kamal et al. 2020). Cellular senescence is a permanent form of cell cycle arrest that halts cell proliferation, but the cells remain viable. The irreversible growth arrest in senescent cells is governed by intrinsic and extrinsic factors, which include pathways for the cyclin-dependent kinase inhibitors (CdkIs) such as p16 and p21 (Regulski 2017). Hallmarks of cellular senescence include increased expression of senescence-associated β-galactosidase activity, p16, p53, and p21. In addition, higher levels of DNA damage, hence, γ-H2AX, are also notable (Noren Hooten and Evans 2017).

Albeit the BBB may be more vulnerable to systemic inflammation in neurological disease (Varatharaj and Galea 2017), BBB disruption also takes place in healthy aging individuals with no apparent disease state (Erickson and Banks 2019). During the adult period and the event of aging, BBB dysfunction emerges and is accompanied by inflammation and loss of paracellular tightness, with no leukocyte recruitment. Typically, the cellular damage that occurs when the BBB is damaged leads to increased apoptosis or cellular senescence, which contributes to aging (Erdö et al. 2017). Still, even though it is known that BBB disturbance takes place in both healthy aging and diseased states accompanied by aging, studies on the association of aging and BBB compromise are scarce.

Over the years, the mouse has remained as the mammalian model of choice for disease modeling in animals. Not only is there a highly conserved genetic homology between mice and humans, but the mouse is easy to breed and employ in the laboratory (Gurumurthy and Lloyd 2019). Considering this, in vivo studies done in mice need to have counterparts in vitro to enable an in-depth probing of cellular as well as molecular mechanisms of diseases. Research on BBB aging is currently limited to the use of patient material or ex vivo material derived from mouse models (Vafaie et al. 2014; Meyer et al. 2016; Yamazaki et al. 2016; Balint et al. 2019). For this reason, a mechanistic follow-up of the underlying senescence-dependent pathways leading to loss of cellular function is not possible.

Since no in vitro senescence induction at the BBB has been attempted thus far, and it being a necessary tool to study the pathophysiology of aging-related diseases involving compromised BBB function, we herein investigate the expression of senescent markers in relation to BBB integrity in vitro. The purpose of this study is to report the association of senescent markers expression to decline of BBB integrity using our BBB model cell line. The immortalized microvascular endothelial cell lines cEND and cerebEND generated in our laboratory (Förster et al. 2005; Silwedel and Förster 2006; Helms et al. 2016) are both established BBB models. They have been used for drug transport studies and as an in vitro model of stroke (Neuhaus et al. 2012, 2014; Salvador et al. 2015; Shityakov et al. 2016; Burek et al. 2019; Ittner et al. 2020). Hence, they are both suitable models for in vitro studies of senescence at the BBB. Nonetheless, our study is limited to the use of the endothelial cells generated in our laboratory, since this is a preliminary attempt at a model of the senescent BBB.

## Materials and methods

### Cell culture

Reagents for cell culture were obtained from Sigma-Aldrich unless otherwise indicated. Cells were cultured in a 37 °C incubator (Steri-Cult 200, Forma Scientific). Primary murine brain (Pelo Biotech) and cerebellar (cerebEND) (Silwedel and Förster 2006) microvascular endothelial cells were cultured in Dulbecco’s Modified Eagle’s Medium (DMEM) supplied with 50 U/ml penicillin/streptomycin and 10% fetal calf serum. Additional supplements were added for culturing cerebral murine microvascular brain endothelial cells (cEND) (Förster et al. 2005; Burek et al. 2012): 2% l-Glutamine, 2% MEM, 2% non-essential amino acids, and 2% Na-pyruvate. Primary cells and cells between passages 22–70 were used in experiments. They were seeded in 6-well plates (Greiner Bio-One GmbH) with or without cover slips with a density of 40,000 cells/cm^2^ and cultured until 95% confluent prior to use in experiments.

### Induction of senescence

Cells between passages 22–70 were induced to senescence by treatment with 100, 150, and 200 µM H_2_O_2_ (Sigma-Aldrich) for 90 min. Afterwards, the medium was changed and the cells were incubated for 24 h at 37 °C (Chen et al. 2007). Next, they were washed twice with phosphate-buffered saline (PBS) (Sigma-Aldrich) prior to further processing for permeation assay, immunofluorescent staining, and Western blot analysis.

### Immunofluorescent staining and microscopy

Cells grown on 20 mm diameter cover slips were fixed in methanol for 20 min at −20 °C. Next, they were washed thrice with PBS for 10 min each and blocked with 5% donkey serum (EMD Millipore) in PBS for 1 h at room temperature. Primary antibodies (anti-p15/16 conjugated to Alexa Fluor 555 diluted 1:500, Santa Cruz Biotechnology; anti-p21 conjugated to Alexa Fluor 488 diluted 1:500, Santa Cruz Biotechnology; anti-claudin-5 conjugated to Alexa Fluor 488 diluted 1:500, Thermo Fisher Scientific; anti-ZO-1 conjugated to Alexa Fluor 488 diluted 1:500, Thermo Fisher Scientific; anti-β-gal diluted 1:500, Cell Signaling; anti-γ-H2AX diluted 1:500, Merck Millipore; anti-Ki67 diluted 1:500, Santa Cruz Biotechnology; anti-GLUT-1 diluted 1:500, EMD Millipore; and anti-VE-Cadherin diluted 1:200, Santa Cruz Biotechnology) were diluted in PBS with 5% donkey serum and allowed to bind overnight at 4 °C. All of the commercial antibodies used have been validated by the manufacturer as given in the specification sheets. Cells were then washed thrice as previous before addition of the secondary antibody (anti-rabbit Alexa Fluor 555, anti-mouse Alexa Fluor 488, anti-goat Alexa Fluor 488, each one diluted 1:400, Thermo Fisher Scientific), if needed, which was allowed to incubate for 1 h at room temperature. Cells were washed again as previous before mounting on glass slide with Fluoroshield + DAPI (Abcam). Samples were viewed using the Leica DM13000B microscope with a Leica DFC 450C camera. The following image acquisition conditions were used with the aid of the Leica V4.13 software: exposure time—11.4 to 287.2 ms for DAPI, 6.5 s for p16 and p21, 4 s for claudin-5, ZO-1, β-galactosidase and γ-H2AX, 2 s for GLUT-1, and 12 s for VE-Cadherin; gain—1×; and gamma—1.13. Quantification of β-gal was carried out by counting positively stained cells and calculating the percentage compared to the total cell number. The ImageJ software (NIH, USA) was used for cell counting.

### Western blot analysis

Cells were lysed with RIPA buffer (50 mM Tris pH 8.0, 150 mM NaCl, 0.1% SDS, 0.5% sodium deoxycholate, 1% NP40) containing protease inhibitor cocktail (Roche). They were then sonicated (Bandelin SONOPULS) and mixed with Laemmli buffer containing 5% β-mercaptoethanol. After denaturation, they were run through a 10–12% SDS PAGE mini gel and blotted overnight using a Mini Trans-Blot Electrophoretic Transfer Cell (Bio-Rad). Subsequently, the membrane was blocked in 5% non-fat dry milk (Carl Roth) and probed with the primary antibody anti-claudin-5 (1:500, Thermo Fisher Scientific) or anti-γ-H2AX (1:500, Merck Millipore) and horse radish peroxidase-conjugated anti-β-actin (1:25,000, Sigma-Aldrich) as endogenous control overnight, followed by secondary antibodies anti-mouse/rabbit (1:3000, Roche Lumi Light Plus and Cell Signaling Technology). The aforementioned commercial antibodies used have been validated by the manufacturer as given in the specification sheets. Detection was carried out using an enhanced chemiluminescence solution (Whitehead et al. 1979) and viewed with Imagen Flour Chem FC2 (Cell Biosciences) with the AlphaView Software (Version 1.3.0.7, Innovatech Corporation). Densitometric analysis was carried out using the Image J software (NIH, USA).

### Transendothelial electrical resistance (TEER) measurement

Upon treatment of cells grown on transwell-inserts (Corning) with a pore diameter of 0.4 μm, TEER was measured using the volt-ohm meter device (EVOM, World Precision Instruments). Blank filters were used as internal control.

### Endothelial permeability measurement

Cells grown on transwell-inserts were placed into a plate with 1.5 ml HEPES-buffered Ringer’s solution (pH 7.4). 1 mM fluorescein (Sigma) was dissolved in the aforementioned solution, of which 500 µl was added to the upper compartment of the insert. For a total duration of 1 h, the inserts were transferred into new wells with fresh solution every 20 min. Each time, aliquots of the solution were collected from the receiver compartment. In the same way, aliquots were taken from the donor compartment at the beginning and end of the assay. Samples were measured using the Tecan Microplate Reader (Thermo Fisher Scientific) at 490/516 nm. Three inserts were measured for each treatment condition, including an insert containing no cells. Permeability coefficient was calculated as previously described (Curtaz et al. 2020).

### Statistical analysis

Statistical significance was determined by unpaired t test or one-way ANOVA, where *p* < 0.05 is considered significant using the Graph Pad Prism 6 program (Graph Pad Software Inc).

## Results and discussion

### H_2_O_2_ induces senescence marker expression

Cultured cells that undergo senescence are not able to undergo DNA synthesis, in the same way as quiescent cells. Associated with the senescent phenotype is elevated expression of senescence-associated beta-galactosidase (β-gal). The presence of this biomarker is, however, independent of DNA synthesis, which thus helps to distinguish senescent from quiescent cells (Itahana et al. 2007).

**Fig. 1:**
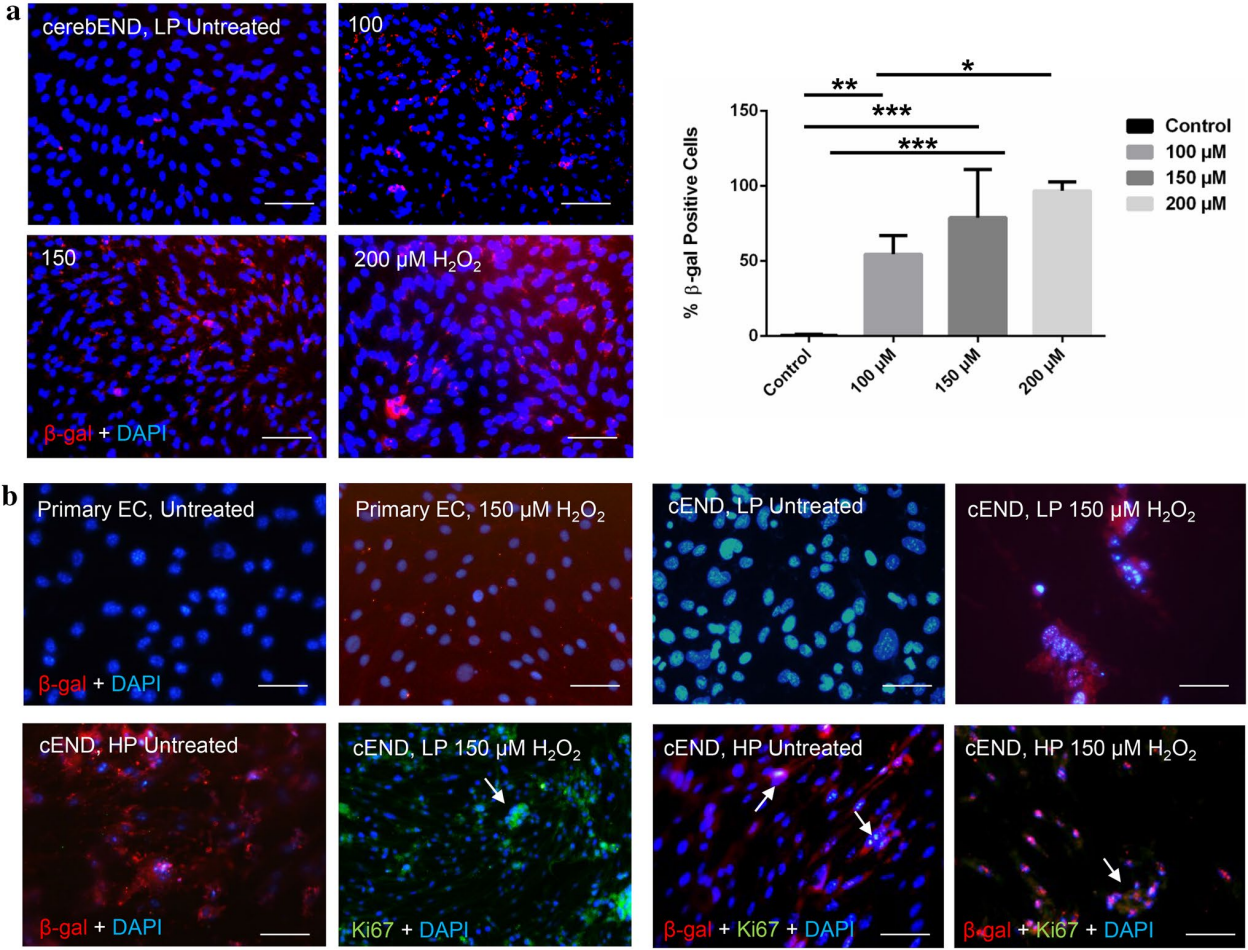
Alterations in endothelial cells brought about by aging and senescence induction. **a** Increased concentration of H_2_O_2_ increases beta-galactosidase (β-gal) expression in cerebellar endothelial cells (cerebEND). Magnification 10 × . Representative images from at least three independent experiments. Scale bar 200 µm. Increase in β-gal-positive cells is significantly different for all H_2_O_2_ concentrations used, compared to control (*p* values: 100 µM =  <0.0001, 150 µM = 0.0028, 200 µM =  <0.0001). **b** Untreated primary endothelial and low-passaged cerebEND cells do not express β-gal, while high-passaged cEND cells do. Treatment of the various cells, both low- and high-passaged, with 150 µM H_2_O_2_, leads to β-gal expression. Both untreated and H_2_O_2_-treated cEND cells express β-gal as well as Ki67. *Arrows* indicate staining of Ki67. Magnification 40×. Scale bar 50 µm

One factor that brings about senescence is oxidative stress, which could be induced in vitro via hydrogen peroxide (H_2_O_2_), an oxidant produced in aerobic metabolism and inflammation (Chen and Ames 1994). Hence, we induced senescence in our murine brain endothelial cell lines as well as primary endothelial cells (EC) via H_2_O_2_ treatment. We observed that increasing concentrations of H_2_O_2_ (100–200 μM) led to an increased amount of β-gal-positive cells in cerebellar endothelial cells (cerebEND), whereby all concentrations used were significantly different compared to control (Fig. 1a).

Likewise, high-passaged cerebral endothelial cells (cEND) stained positive for β-gal, while untreated primary EC as well as low-passaged ones did not. However, treatment of the low-passaged cEND and primary ECs with H_2_O_2_ rendered positive staining for β-gal (Fig. 1b). Altogether, this posits that older cells in culture (i.e., late passages) undergo senescence due to oxidative stress. This could be replicated in early passages of cells by inducing senescence using H_2_O_2_. Meanwhile, positive staining for the proliferation marker Ki67 was observed in both untreated and treated high-passaged cells (Fig. 1b). Senescent cells have elevated levels of β-gal as well as persistent DNA damage response distinguishing them from non-proliferating cells (Campisi and d’Adda di Fagagna 2007; Kuilman et al. 2010).

**Fig. 2:**
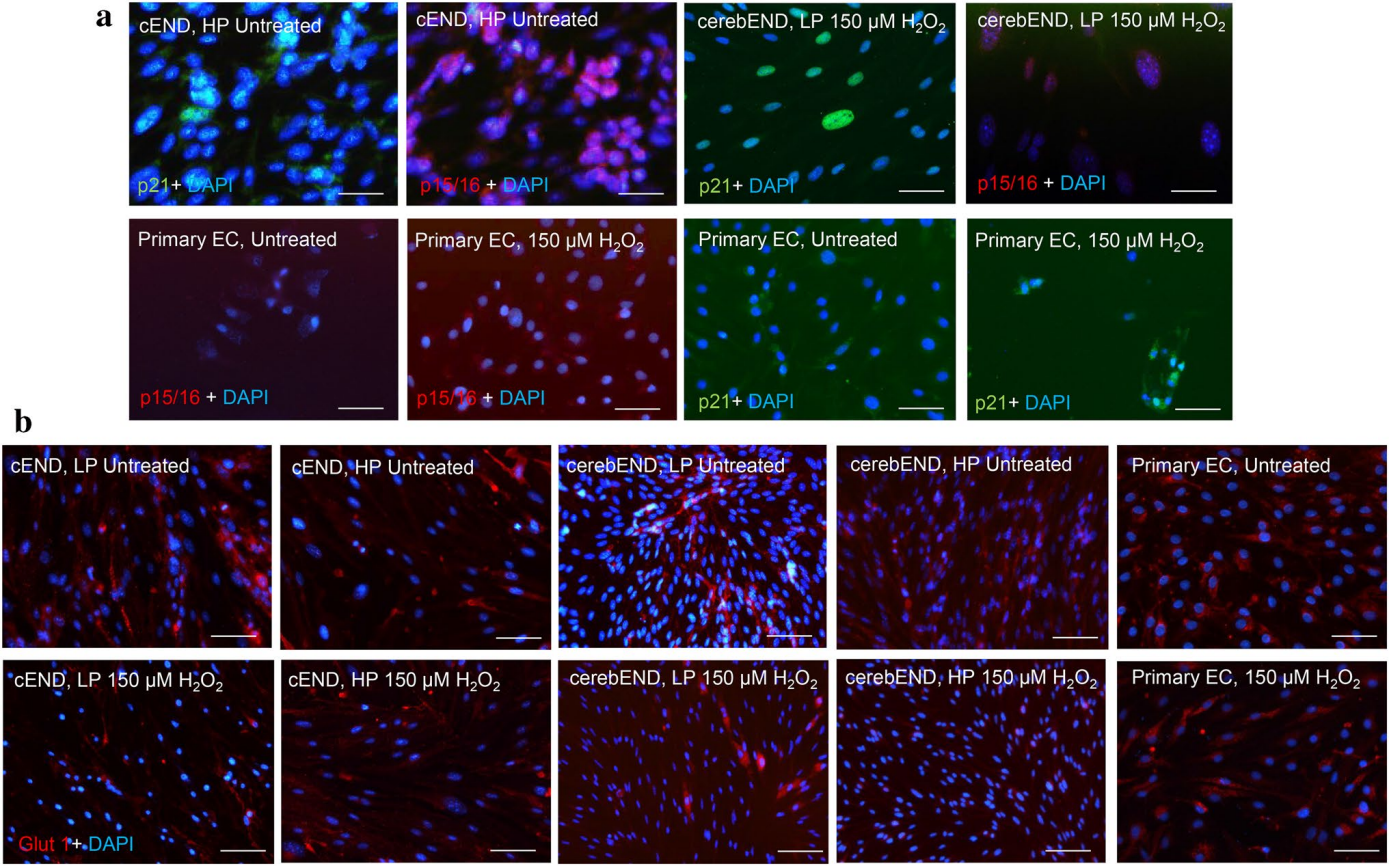
Effects of senescence induction on expression of senescence markers and glucose transporter-1. **a** High-passaged untreated cEND cells express p21 and p16. Low-passaged cerebEND cells treated with 150 µM H_2_O_2_ induced p21 and p16 expression. Meanwhile, primary cells do not express both markers if left untreated. **b** Antibody against glucose transporter (glut)-1 renders staining in both treated and untreated primary, cEND and cerebEND cells of both low and high passages. Reduction of staining is visually observed in cEND and cerebEND upon treatment with 150 µM H_2_O_2_, but not in primary endothelial cells. Representative images from at least three independent experiments. Magnification 40×. Scale bar 50 µm

In our BBB model, assessment of β-gal, p16, and p21 demonstrates their expression in high-passaged cells but not in lower passaged ones. Untreated cEND cells of high passage as well as treated low passage showed both p16 and p21 staining. Meanwhile, primary ECs stained positive for both, only upon administration of 150 µM H_2_O_2_ (Fig. 2a).

Both p16 and p21 are components of the tumor suppressor pathways governed by p53, a transcriptional regulator disrupted in cancer (van Deursen 2014). p16 is one of the most useful in vivo markers of senescence. As a regulator of the cell cycle, p16 is involved in limiting G1 to S-phase progression. Its expression is undetectable in healthy young tissues, but increases notably in many aging or injured tissues (Liu et al. 2019). Meanwhile, p21, directly induced by p53, functions as cell cycle inhibitor arresting the G1 phase and acts as an anti-proliferative effector (Georgakilas et al. 2017). The presence of these markers in high-passaged cells but not in the lower passaged ones confirms aging endothelial cells to be carrying characteristics of senescence.

### Senescence induction alters expression of transporter and junctional proteins

Endothelial cells are highly glycolytic, even in quiescent conditions. For this reason, they require constant uptake of glucose, which majorly takes place via the glucose transporter (GLUT)-1 (Fitzgerald et al. 2018). To examine the effects of senescence induction to the expression of GLUT-1 in our model, we conducted immunofluorescent staining. In our observation, expression of GLUT-1 was evident in both treated and untreated primary, cEND and cerebEND cells of both low and high passages. A decreased and more dispersed staining could be visually observed in treated cEND and cerebEND cells. However, GLUT-1 staining in primary ECs appeared unaltered (Fig. 2b). This result is in accordance to a study where measurement of GLUT-1 expression in brain-derived endothelial cells using median fluorescence intensity showed a significant decrease among patients with Alzheimer’s disease compared to controls, although there was no reduction in cell numbers (Vogelsang et al. 2018).

**Fig. 3:**
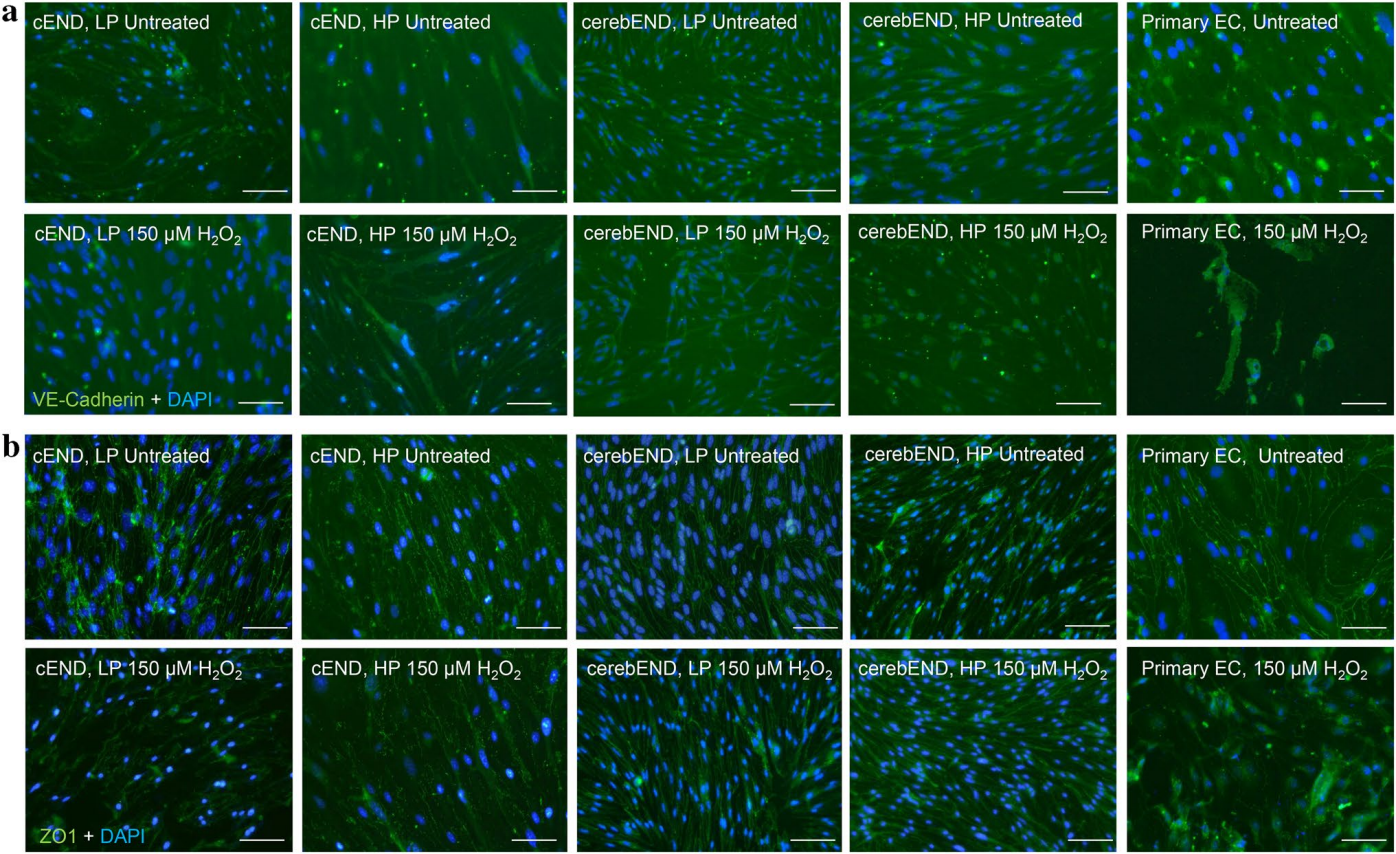
Changes in expression of adherens and tight junctions after senescence induction. **a** VE-Cadherin is localized along the cell membrane of untreated low-passaged cEND and cerebEND cells. Untreated high-passaged cEND, primary endothelial as well as treated cells show dispersed staining. **b** ZO-1 outlines the borders of untreated cEND and primary endothelial cells. When 150 µM H_2_O_2_ was administered, both cells lost the typical border staining of ZO-1. On the other hand, cerebEND cells appear the same with or without treatment. Magnification 40 × . Scale bar 50 µm

**Fig. 4:**
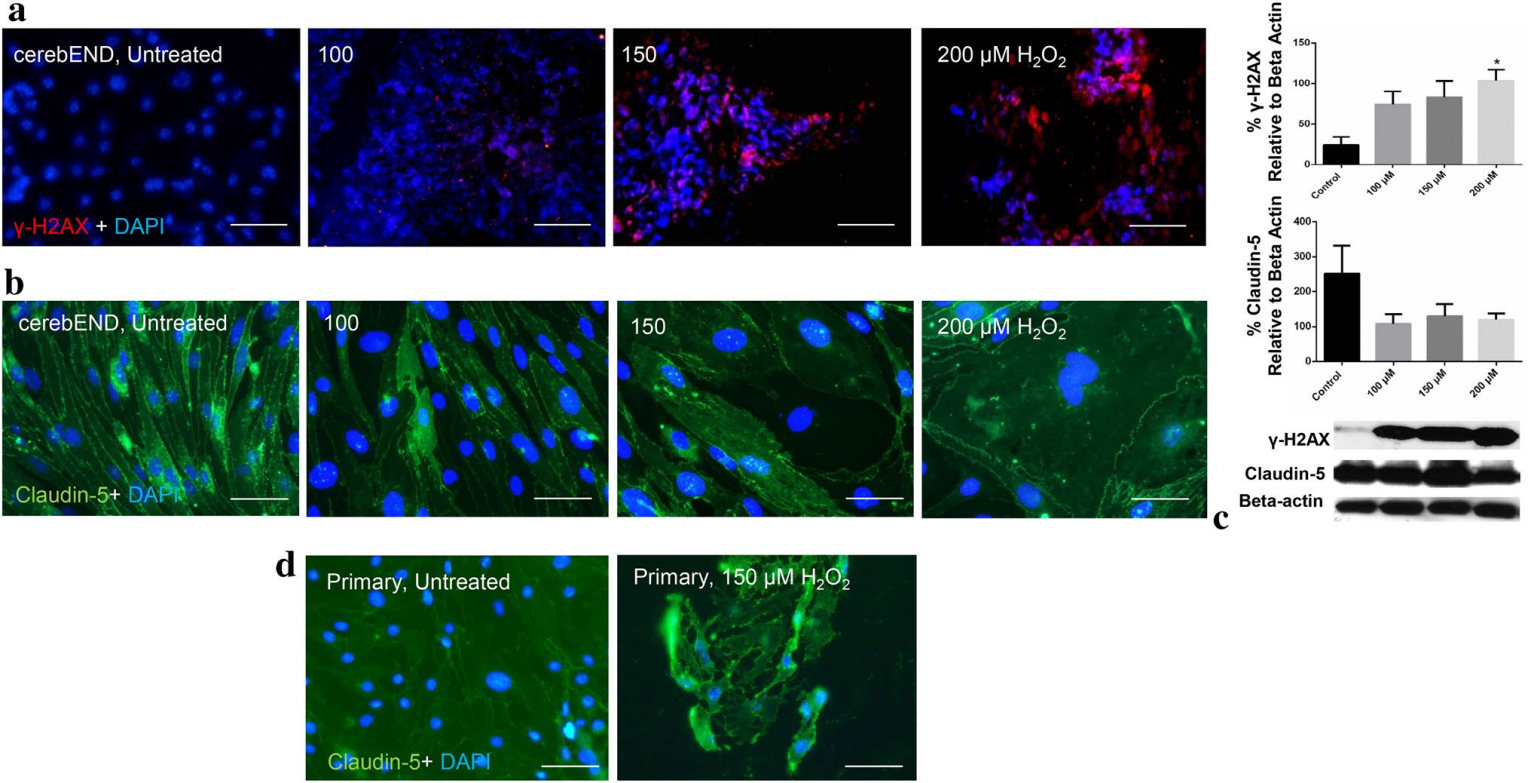
Effects of senescence induction on DNA damage and tight junction integrity. **a** Claudin-5 delocalizes in primary endothelial cells upon treatment with 150 µM H_2_O_2_. **b** With higher H_2_O_2_ concentration, claudin-5 expression decreases in cerebEND cells, while **c** γ-H2AX expression increases. **a–c** Immunofluorescence staining of representative images from at least three independent experiments. Magnification: **a**, **b** = 40 × , **c** = 10 × . Scale bar: **a**, **b** = 50 µm, **c** = 200 µm. **d** Western blot analysis. Claudin-5 expression decreased with H_2_O_2_ treatment. γ-H2AX expression increases with higher H_2_O_2_ concentration showing 200 µM as being significantly different from control (*p* = 0.0336). Densitometric values were analyzed from three independent experiments

Considering that tight- and adherens junction proteins are major determinants of BBB integrity, we visually appraised the staining pattern of VE-Cadherin, ZO-1, and claudin-5 in our model. The distribution of VE-Cadherin at cell–cell contacts of untreated cEND, cerebEND and primary ECs showed a regular pattern, with the exception of high-passaged cEND cells, compared to those treated with 150 μM H_2_O_2._ High-passaged cEND cells appear similar to the treated ones (Fig. 3a). This result agrees with previous observation in human umbilical vein endothelial cells (HUVEC) treated with conditioned medium from senescent cells which reported an irregular VE-Cadherin disposition inside the cells (Wong et al. 2019). In the same way, the staining pattern of ZO-1 in treated cEND and primary ECs is disrupted compared to untreated control. However, senescence induction with 150 μM H_2_O_2_ did not alter the morphology of ZO-1 staining pattern in cerebEND cells (Fig. 3b). Accordingly, decreased expression of claudin-5 was noted in H_2_O_2_-treated primary and cerebEND cells (Fig. 4a, b). Of note, senescence-induced cerebEND cells were enlarged and had bigger nuclei, giving rise to a disrupted endothelial cell morphology. Normal control cells were spindle-shaped (Fig. 4b). It is known that adherens and tight junctions are disrupted in senescent cells, leading to BBB dysfunction (Krouwer et al. 2012).

In conjunction, we examined DNA damage, by assessing the expression of DNA-double-strand break biomarker γ-H2AX. This histone octamer component in nucleosomes was detected among senescence-induced cEND cells but not in the control (Fig. 4c). Western blot analysis confirmed this by showing a trend of increasing γ-H2AX expression, with the highest concentration of 200 μM being significantly different from the control (*p* = 0.0336). In conjunction with the immunofluorescence staining observation, claudin-5 levels showed a decreasing trend with increased H_2_O_2_, as compared to control, albeit not statistically significant (Fig. 4d).

Several studies indicate the association of senescence with compromised BBB integrity. For instance, in an in vitro model constructed with senescence-induced primary endothelial cells, pericytes, and astrocytes, tight junction structure and barrier integrity were significantly impaired (Yamazaki et al. 2016). These results, together with our preliminary findings, indicate that accumulation of senescent vascular cells is associated with compromised BBB integrity, providing insights into the mechanism of BBB disruption related to biological aging.

### Senescence induction changes barrier permeability and integrity

**Fig. 5:**
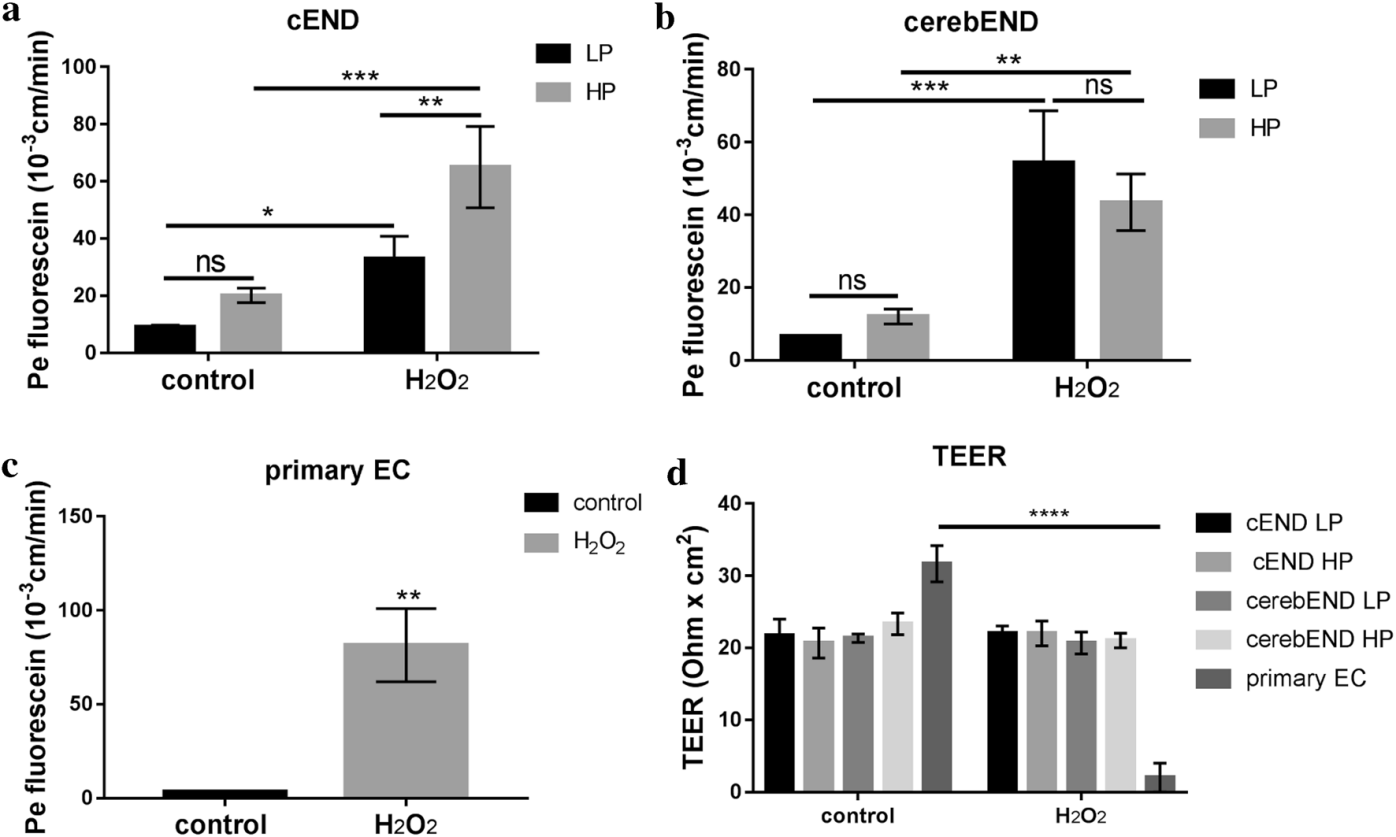
Permeability and integrity of the cells are altered by senescence induction with 150 µM H_2_O_2_. **a**–**c** Fluorescein permeability is significantly increased in cells treated with 150 µM H_2_O_2_. **d** Transendothelial electrical resistance (TEER) of senescence-induced primary endothelial cells significantly declined albeit there is no change among cEND and cerebEND cells. Values were analyzed from triplicate wells

To evaluate the effects of senescence induction to barrier integrity, the permeation of fluorescein through both treated and untreated low and high-passaged cells was determined. Untreated high-passaged cEND and cerebEND cells exhibited a trend of slightly greater permeability compared to low-passaged cells, albeit not significant (Fig. 5a, b). On the other hand, treated high-passaged cEND cells were significantly more permeable to fluorescein compared to low-passaged ones (Fig. 5a). In the same way, senescence induction with H_2_O_2_ significantly increased permeation in both cEND and cerebEND cells regardless of being low or high passages (Fig. 5a, b). Fluorescein permeation significantly increased in primary ECs due to senescence induction (Fig. 5c). Nonetheless, H_2_O_2_ appears to impart no effect on the integrity of both cEND and cerebEND cells as observed through transendothelial electrical resistance (TEER) measurements (Fig. 5d). However, TEER of primary ECs significantly decreased after application of H_2_O_2_ (*p* < 0.0001). The TEER values of both cEND and cerebEND cells, regardless of passage, appear to be significantly lower than that of primary endothelial cells. In vivo, the rat BBB has a recorded TEER of 5900 Ω cm^2^ (Butt et al. 1990). This value is, however, difficult to achieve among in vitro BBB models. For instance, primary human brain endothelial cells (HBMECs) and immortalized human brain endothelial cell line hCMEC/D3 both have a TEER value of 100 Ω cm^2^ (Daniels et al. 2013), whereas the mouse brain endothelial cell line bEND.3 co-cultured with C8-D1A astrocytes in a transwell system demonstrated a TEER value of 25 Ω cm^2^ (Booth and Kim 2012). Thus, in general, although there appears to be no significant difference in the TEER values of treated and untreated cerebEND and cEND cells, the results we obtained in this study are indicative that senescence induction with H_2_O_2_ results to an altered permeability and compromised barrier integrity.

Overall, senescent marker expression varied between primary ECs as well as the low- to high-passaged and senescence-induced endothelial cell lines used in our model. Moreover, expression of tight junction protein such as claudin-5 decreased in cells expressing senescent markers. Thus, these initial findings could draw out a possible relationship between senescence and BBB integrity and warrant further investigation. Suitable in vitro models of aging endothelial cells are currently lacking. Although Yamazaki and colleagues (2016) demonstrated that the accumulation of senescent vascular cells is associated with compromised BBB integrity, the in vitro BBB model is composed of primary cultures of mouse endothelial cells and pericytes, as well as astrocytes. Despite the fact that primary cells represent what is occurring in vivo more than immortalized cell lines, the latter are more cost-effective and easier to handle. Moreover, they provide a pure population of cells, which lead to a more stable platform and reproducible results (Kaur and Dufour 2012). Hence, our proposed use of the two immortalized murine cell lines we generated in our laboratory, namely, cEND and cerebEND cells as basic in vitro models systems for the senescent BBB, is advantageous. With this in mind, this initial attempt to provide an in vitro model of the senescent BBB may prove useful for looking into mechanistic insights of the aging BBB. Further investigations, which include additional cells of the BBB, like pericytes and astrocytes, should be carried out in the future.

## Data Availability

Data and materials pertaining or in relation to the findings presented herein are available upon request.

## Abbreviations

BBB: Blood–brain barrier
β-gal: Beta-galactosidase
CdKIs: Cyclin-dependent kinase inhibitors

## Code availability

Software availability available upon request.
